# Teacher-Delivered Mental Health Interventions: Promises, Challenges, and Recommendations for Future Directions

**DOI:** 10.1007/s10488-025-01467-6

**Published:** 2025-08-28

**Authors:** Gwendolyn M. Lawson, Andrew Orapallo, Golda S. Ginsburg, Avery Brewton, Courtney N. Baker, Gazi Azad

**Affiliations:** 1 Department of Child and Adolescent Psychiatry and Behavioral Sciences, Children’s Hospital of Philadelphia, Philadelphia, PA USA; 2 Department of Psychiatry, Perelman School of Medicine, University of Pennsylvania, Philadelphia, PA USA; 3 Department of Psychiatry, University of Connecticut School of Medicine, Farmington, CT USA; 4 Coalition for Compassionate Schools, New Orleans, LA USA; 5 Department of Psychology, Coalition for Compassionate Schools, Tulane University, New Orleans, LA USA; 6 Heilbrunn Department of Population and Family Health, Mailman School of Public Health, Columbia University Irving Medical Center, New York, NY USA

**Keywords:** Teachers, School mental health, Multitiered systems of support, Prevention

## Abstract

Schools are a key setting where services to support youth mental health can occur, and teachers are important for students’ social, emotional and behavioral well-being. Teacher-delivered mental health prevention and intervention programs offer an opportunity to integrate mental health support meaningfully into students’ everyday lives, as well as expand the reach and impact of mental health services. This is particularly important given the lack of highly trained mental health providers and the barriers to accessing clinical services. However, teachers are not trained as mental health providers and serve a primarily educational mission, and therefore, there are unique considerations for conceptualizing teachers as individuals who can deliver mental health prevention and intervention programs. The purpose of this paper is to delineate conceptual and practical issues related to utilizing teachers as non-traditional mental health providers including key opportunities and challenges to teacher-delivered mental health interventions. We present four examples of teacher-delivered programs that aim to support student mental health and well-being and use these example programs to illustrate these key opportunities and challenges. We also outline directions for future research, with the ultimate goal of enhancing teachers’ skills and improving youth mental health.

Youth mental health concerns are a pressing challenge in the United States. In 2016, the national prevalence estimate of at least one diagnosed mental health disorder among children and adolescents was 16.5%. Rates of mental health concerns and service needs were exacerbated during the COVID-19 pandemic (Close et al., 2024; Whitney & Peterson, 2019), and remain at an alarmingly high rate. Unfortunately, only about half of youth in need of services receive treatment from a mental health professional (Whitney & Peterson, 2019), with even lower rates among marginalized or low-income youth (Costello et al., 2014). This is not surprising given the shortage of highly trained mental health providers, with only about 14 pediatric psychiatrists and 5 pediatric psychologists per every 100,000 youth (Close et al., 2024).

Task sharing the delivery of youth mental health interventions with non-traditional providers (i.e., individuals without specialty mental health training) has been suggested as a potential solution to address the gap between service needs and treatment access (Kazdin, 2019). Given that child development is impacted by multiple interconnected systems (i.e., families, schools, community, and economic systems; Bronfenbrenner, 2005), recent models of mental health service provision (e.g., Zhou, 2020) highlight the range of adults, beyond highly trained mental health providers, who can support youth mental health. For example, parents and caregivers can deliver caregiver-mediated interventions after training in behavioral parent training programs (Eyberg et al., 2008; Kjøbli et al., 2023); paraprofessional youth mentors can support or even directly deliver mental health care (McQuillin et al., 2022); and mental health interventions can be modified for delivery by child welfare staff (Gopalan et al., 2019). Models that extend the conceptualization of “youth mental health services” beyond traditional clinics and specialty trained providers hold great promise for improving the reach, effectiveness, and impact of the youth mental health care system (Kazdin, 2019). At the same time, there are important considerations about tasking individuals with healthcare responsibilities when they may not have the appropriate training, support, and resources, especially in service delivery systems already tasked with multiple responsibilities (Malterud et al., 2020; Olding et al., 2021).

## Mental Health Services in Schools

Schools are a key setting where services to support youth mental health and well-being can occur (Hoover & Bostic, 2021). Students spend an average of 1,000 h per year in school (National Center for Education Statistics, 2024), making their interactions with peers and educators in this setting crucial for their well-being. Schools increasingly offer embedded or co-located mental health clinics (Hoover et al., 2019) and are among the most common settings for youth to access mental health services (Duong et al., 2021). Moreover, school-based mental health promotion programs, such as social-emotional learning (SEL) programs and school-wide systems such as positive behavior interventions and supports (PBIS), can also be conceptualized as an important part of the continuum of youth mental health services (Hoover & Bostic, 2021).

School mental health services are often guided by a multi-tiered system of support (MTSS) framework (Kern & Rusnak, 2024). Within this framework, universal (i.e., Tier 1) interventions are provided to all students within the school. Tier 1 interventions commonly include SEL programs delivered to the entire class, school-wide behavioral expectations and reinforcement systems, and programs to support school climate. Targeted (i.e., Tier 2) interventions are provided to students in need of additional support, but not necessarily with a mental health diagnosis. Tier 2 interventions are commonly delivered to small groups of students, but also can include brief, individual interventions such as Check-In/Check-Out or Daily Report Cards (Owens et al., 2024). Finally, indicated (i.e., Tier 3) interventions are individualized, more intensive services for students impacted by mental health conditions, such as ongoing individual or family therapy. It is increasingly common for schools – often in collaboration with community partners – to offer mental health prevention and intervention services along this full continuum (Hoover et al., 2019).

### Teacher-Delivered Mental Health Interventions

Although teachers are not traditionally trained as mental health providers, some existing mental health interventions can be delivered by teachers, most frequently at the whole class (i.e., Tier 1) level. A systematic review of 49 school mental health intervention studies found that teachers were actively involved in about 41% of the interventions evaluated and were the sole providers of interventions for about 18%. The majority of the teacher-delivered interventions were Tier 1 interventions (e.g., “Life Skills Training”), but several were also delivered at Tiers 2 or 3 (e.g., “Anger Control Curriculum;” Franklin et al., 2012). To date, evidence regarding the effectiveness of teacher-delivered interventions for student mental health outcomes is mixed.

At Tier 1, meta-analyses of universal SEL programs which generally do not have an explicit mental health focus though often share core components with mental health interventions, tend to find improvements in students’ skills, behavior, and school functioning (Cipriano et al., 2023; Durlak et al., 2011). For Tier 1 interventions more explicitly focused on student mental health, meta-analytic evidence suggests a small impact of teacher-delivered interventions on student internalizing outcomes. Park et al. (2020) found an overall significant effect for internalizing behaviors across whole classroom-based interventions (*d* = 0.22). Similarly, Shelemy et al., 2020 found that students who received teacher-delivered interventions at the universal level showed significant improvements in depression and anxiety symptoms, compared to control conditions, although improvements were only maintained at follow-up for anxiety symptoms, and Hedge’s *g* did not meet the threshold for a small effect size. Additionally, Park et al. (2020) examined studies of student externalizing outcomes and did not find a significant impact of Tier 1 teacher-delivered interventions. However, meta-analyses of classroom-wide behavior management interventions implemented by teachers tend to show small, but meaningful effects in reducing student disruptive and off-task behaviors (e.g., Korpershoek et al., 2016; Smith et al., 2021).

Two of the previously-mentioned meta-analyses also included studies of Tier 2 or Tier 3 teacher-delivered interventions measuring internalizing outcomes (Park et al., 2020; Shelemy et al., 2020). Neither meta-analysis found a significant impact of these “non-Tier 1” teacher-delivered interventions on student internalizing or externalizing outcomes. However, Aldabbagh et al. (2024) conducted a meta-analysis of supports delivered directly to teachers (e.g., training on classroom management interventions) that aimed to benefit teaching practices and/or children ages 2–13 with high levels of externalizing behavior (i.e., ADHD symptoms or conduct problems). They found an overall moderate but significant impact of interventions on reducing student externalizing behavior. Collectively, this evidence suggests that the impact of teacher delivered mental health interventions may be moderated by target population (e.g., students with externalizing vs. internalizing symptoms) and intervention-level factors (e.g., tier of delivery); it also suggests that more research is needed to identify the optimal intervention-level and contextual characteristics for mental health interventions delivered by teachers.

## Current Paper

The purpose of this paper is to delineate the unique considerations related to utilizing teachers as non-traditional mental health providers. We outline issues to consider when conceptualizing teachers in this way, including key opportunities and challenges to teacher-delivered programs to support student mental health. We present four examples of teacher-delivered prevention and intervention programs to illustrate these opportunities and challenges. Finally, we discuss directions for future research.

## Conceptual Issues

Substantial evidence indicates that teachers can have a meaningful effect on students’ social-emotional, behavioral, and educational functioning both concurrently and prospectively (Mashburn et al., 2008; Murray & Greenberg, 2001; Wang et al., 2020). Hoover and Bostic (2021) also point out that much of the education teachers provide as part of their everyday instruction include skills relevant to mental health, such as managing stress, problem solving, working effectively with others, and managing frustration. Furthermore, teachers frequently are responsible for delivering prevention and early-intervention programs to support children’s social, emotional, and behavioral well-being. For example, a large national survey found that over half of teachers reported implementing positive behavior systems or SEL programs or practices (Hamilton et al., 2019). Evidence-based SEL programs, including those delivered by teachers, share many core components (e.g., feelings identification, problem solving) with evidence-based mental health interventions delivered by highly-trained mental health providers (Lawson et al., 2019). However, utilizing teachers as non-traditional mental health providers presents unique opportunities and challenges.

### Opportunities

Intervention models that leverage teachers as non-traditional mental health providers offer important opportunities for both teachers and students. For teachers, the additional training and supports provided when teachers implement a mental health intervention is an important benefit. The high prevalence of mental health disorders presents challenges to teachers because students with mental health conditions require specialized skills to manage their complex social, emotional, behavioral, and educational needs. However, given that teacher training in mental health is limited, teachers often feel ill equipped to provide effective instruction, minimize disruptions, and maintain a positive student-teacher relationship within the context of unmet student mental health needs (Dimitropoulos et al., 2022). Conversely, when teachers are equipped with the appropriate knowledge and skills, this can support their mastery in managing students’ social and emotional challenges. In addition to benefiting students, this also has benefits for teachers as teacher self-efficacy is associated with their own well-being and job satisfaction. (Goddard et al., 2004; Zee & Koomen, 2016).

For students, teacher-delivered interventions, as a complement to mental health interventions delivered by trained mental health providers, have several potential benefits. Utilizing teachers offers promise for integrating mental health intervention more meaningfully into children’s everyday lives (e.g., coping with peer rejection at school lunch), which may effectively improve both mental health and educational outcomes (Atkins et al., 2010; Cappella et al., 2011). Moreover, teacher-delivered interventions can be less stigmatizing than traditional models (Singh et al., 2022), and therefore, may reach Black, Latine, Asian, or other subgroups in which mental health care is more likely to be stigmatized. Teacher-delivered mental health interventions also have the potential to be highly scalable. Given that there are approximately four million teachers in the United States (National Center for Education Statistics, 2024) and interventions can reach entire classrooms of students, teacher-delivered interventions can be accessed by more youth, resulting in the potential for large scale public health impact. This stands in stark contrast to the reach of individual interventions delivered by highly trained mental health providers. Notably, given the ratios of school psychologists, counselors, and social workers per student are far below recommended ratios (Hopeful Futures Campaign, 2022), teacher-delivered interventions may be an important way to make mental health supports more accessible within the context of these workforce shortages. Taken together, there is clear and compelling evidence that teacher-delivered mental health interventions have the potential to be cost-effective, to reduce barriers to care, and to provide access to mental health supports for children who would not otherwise receive them.

### Challenges

There are also important challenges to utilizing teachers as non-traditional providers of mental health interventions. Teachers are primarily trained as educators, not as mental health providers, and they serve a primarily academic mission (Atkins et al., 2017). Consistent with this, studies examining teachers’ perceptions of their roles regarding student mental health generally find that teachers view student mental health as important, but do not view all mental health support tasks as within the scope of their roles (Maclean & Law, 2022; Reinke et al., 2011). For example, Reinke et al. (2011) found that teachers perceived themselves to have a primary role implementing classroom behavioral interventions, but not screening for mental health problems or referring students to services. Teachers also report feeling inadequately prepared to address student mental health needs (Maclean & Law, 2022; Reinke et al., 2011) and express concerns about working outside their scope of practice (Dimitropoulos et al., 2022). Indeed, asking teachers to deliver specialized mental health interventions that fall outside the bounds of their professional competence would raise a number of ethical concerns, including dual relationships and potential misrepresentation of professional qualifications. Therefore, any mental health supports provided by a teacher must be delivered within their role as a teacher, not as a therapist or other specialized mental health professional. Further, teachers typically lack training to handle crisis situations such as suicidality (Nadeem et al., 2011), so any teacher-delivered interventions must consider these boundaries of professional competence.

Moreover, teachers, especially those in underfunded schools, are often not provided the necessary resources for their primary educational mission (Eiraldi et al., 2015), and therefore, may not have the resources or bandwidth to address the mental health needs of their students. Rates of job-related stress, burnout, and turnover among teachers are already high (Steiner & Woo, 2021), particularly in special education (Hester et al., 2020). Consistent with theoretical models of burnout (Bakker & Demerouti, 2017), there are concerns that conceptualizing teachers as non-traditional mental health providers, especially without adequate support and resources, could exacerbate these issues. Furthermore, teacher-delivered mental health interventions can take time away from academic instruction, and could lead to role confusion with related service providers (e.g., school psychologists, counselors, and social workers) who are specifically trained to provide mental health supports. Indeed, there are possibilities of harm to teachers (e.g., burnout, turnover), students (e.g., iatrogenic mental health impacts, reduced time for academic instruction), and the broader school community (e.g., role confusion) from asking teachers to deliver mental health interventions that are inappropriate for their training, mission, and context.

Given these opportunities and challenges, it is clear that teacher-delivered mental health interventions must be designed and implemented in ways that capitalize on the potential of involving teachers in mental health supports, while also mitigating the potential challenges of doing so. This requires carefully considering teachers’ training, roles, and the classroom context so that teacher-delivered interventions benefit both teachers and students, avoid ethical concerns, and integrate effectively with other school-based mental health professionals (e.g., school psychologists, counselors, social workers).

## Example Programs

To illustrate these issues, we present four examples of programs in which teachers deliver interventions to support student mental health. We intentionally selected these examples to cover four distinct areas of mental health (i.e., traumatic stress, attention deficit/hyperactivity disorder, anxiety, autism). In contrast with traditional SEL or classroom management interventions, each of these example programs has an explicit focus on a specific mental health clinical area. We also selected examples that are delivered across a range of tiers (i.e., Tiers 1 through 3). We note, however, that these interventions are not meant to replace specialized mental health services by school-based mental health providers and are not psychotherapy, but rather are designed to both enhance the capacity of teachers and support students in their natural environments.

Given the relative novelty of teacher delivered-mental health interventions, we selected example programs that have been fully developed and implemented, but are in relatively early stages of evaluation and dissemination. An overview of each example program, including the mental health area of focus, program context, target grade range, and tier of delivery is displayed in Table 1. For each program, we describe the model and theory of change, the current context of implementation, and current evidence of effectiveness. We also highlight key opportunities and challenges that arose during the implementation of each of these programs to date.

**Table 1:** Case examples of Teacher-Delivered prevention and intervention programs

Example Program	Mental health focus area	Program Context	Target Grade Range	Tier of delivery	Key teacher-delivered intervention components	Key training components and other implementation strategies
Safe Schools NOLA (SSNOLA)	Traumatic Stress	Developed with five schools and implemented with 6 additional schools in New Orleans charter schools and networks	Grades K-8	Tier 1	Three trauma-informed classroom engagement strategies: -predictable classroom routines -de-escalation and emotion regulation -supportive relationships	-Full-school trauma training -Three teacher skill-building trainings -Classroom observations and coaching -Consultation with school leadership -Peer support team -Some ability to screen and refer for services for PTSD and psychological distress
Positive Behavior Management Toolkit (PBMT)	ADHD	Developed within schools in Philadelphia	Grades K-5 focus; additional exploratory work in grades 6–8	Tiers 1 and 2	-Behavior-specific praise -Precorrections -Calm behavior-specific corrections -Daily behavior reports	-Resource library (i.e., written documents, videos, tangible resources) -Text-message reminders -Brief, biweekly consultant meetings
Teacher Anxiety Program for Elementary Students (TAPES)	Anxiety	Elementary Schools in New England	K-5	Tier 2	*Five Meetings*: Psychoeducation -Relaxation skills -Behavioral Exposure (i.e., facing fears) -Coping Thoughts -Relapse Prevention	-Six-hour interactive training -Intervention manual and handouts, videos -Trouble shooting guide -Weekly Coaching
Partners in School	ASD	Developed in partnership with Philadelphia, Baltimore, and New York City School Districts. Implemented in NYC schools.	Preschool, Pre-Kindergarten, K-5	Tiers 2 and 3	15 EBPs available for use, but each student intervention plan is usually comprised of 3–5 EBPs. All EBP plans use visual supports and reinforcement strategies.	-Communication skills training -Problem-solving consultation -All intervention materials -Micro-coaching

ADHD = Attention-Deficit/Hyperactivity Disorder; ASD = Autism Spectrum Disorder; EBP = Evidence-Based Practice; K = Kindergarten; PTSD = Post-Traumatic Stress Disorder

### Safe Schools NOLA (SSNOLA)

#### Brief Description and Mental Health Target

Safe Schools NOLA (SSNOLA; Overstreet & Baker, 2022) is a one-year Tier 1 intervention that aims to build individual student competencies through staff training and the development of organizational infrastructure in K-8 schools. SSNOLA, like other trauma-informed approaches (Cole et al., 2013; SAMHSA, 2014), views students as well as school staff in holistic and connected ways, with the goals being to (1) raise awareness of the common experience of stress and trauma, (2) develop an understanding of how it impacts individuals and – in school – their ability to learn, (3) offer a safe space for healing and learning, and (4) prevent further harm. The overarching aims of the intervention are to reduce the negative impacts of traumatic stress exposure among students and school staff and to improve overall school psychological and physical safety. SSNOLA offers schools a framework to achieve these goals, which was adapted from widely used guides on trauma-informed approaches (Cole et al., 2013; SAMHSA, 2014) and pilot tested through university-school partnerships (McIntyre et al., 2019; Wittich et al., 2020).

#### Program Components and Theory of Change

SSNOLA (see Overstreet & Baker, 2022 for a more detailed description) begins with an intensive, full-school professional development training focused on the first two goals: raising awareness of the prevalence of traumatic stress and developing an understanding of how it manifests in individuals, specifically in schools. The purposes of this training are to provide foundational psychoeducation designed for non-mental health providers, build a shared understanding of trauma and its impacts, develop buy-in, and foster a sense of mutual purpose and community, all toward the long-term goal of driving systems change. Spread across the school year, school staff engage in additional activities to address the third and fourth proximal goals – create a safe place for healing and learning, and take action to prevent further harm. Specifically, school staff participate in three skill-building professional development trainings on (a) predictable classroom routines, (b) de-escalation and emotion regulation, and (c) supportive relationships. Simultaneously, school leaders gather needs assessment data and engage in consultation to develop an action plan with the goal of aligning the school’s policies, practices, and procedures with trauma-informed approaches. Implementation is further supported by tiered coaching and peer support specialists. A social justice framework is explicit across all activities (Davis et al., 2022). The theory of change for SSNOLA emphasizes that student impacts are a result of staff and system changes. Specifically, SSNOLA is thought to build school staff knowledge, attitudes, and behavior favorable to trauma-informed care through professional training (Ajzen, 1991; Kirkpatrick, 1967) and embed these workforce changes within a facilitative and supportive school environment (Fixsen et al., 2009).

#### Empirical Evidence of Effectiveness

SSNOLA was evaluated within five K-8 urban schools serving predominantly low-income and mostly Black youth using a multiple baseline experimental research design (Overstreet & Baker, 2022). Teachers and school staff reported being satisfied (87%) and finding SSNOLA to be both acceptable (85%) and feasible (75%). Educators also showed gains in knowledge on a quiz-style measure (*d* = 1.16). Given that SSNOLA is a systems change intervention, all other study outcomes were evaluated in terms of intervention year 1 relative to baseline as well as the average effect during the subsequent 1–2 years relative to baseline. Educators self-reported more favorable attitudes toward implementing trauma-informed approaches (*d*_Yr1_ = 0.38, *d*_Yrs2−3_ = 0.46). Overall, classroom observations showed more positive behavior support and student engagement in the classroom (*d*s_Yr1_ = 0.51-1.24, *d*s_Yrs2−3_ = 1.54–2.95). School staff perceived increases in their school’s capacity to implement trauma-informed approaches (*d*_Yr1_ = 0.58, *d*_Yrs2−3_ = 0.80). Overall, educators reported improvements in school safety, relationships, and climate (*d*s_Yr1_ = 0.30-0.50, *d*s_Yrs2−3_ = 0.71-0.89) and less disruptive behavior in the classroom (no effect for year 1, *d*_Yrs2−3_ = −1.01). Using administrative data, a trend toward fewer out-of-school suspensions was also apparent. However, teachers did not report improvements in aggression and victimization, students did not report any changes in school climate, and, contrary to hypotheses, reported a small increase in aggression and victimization (*d*s_Yr1_ = 0.10-0.14, *d*s_Yrs2−3_ = 0.15-0.27).

#### Opportunities and Challenges

These findings suggest that SSNOLA offers a promising framework for installing trauma-informed approaches in K-8 schools, though further research is necessary to refine the model. Several implementation challenges and opportunities emerged. First, teachers need sufficient personal supports, such as self-efficacy, and system-level supports, such as a supportive implementation climate, to implement the model (Wittich et al., 2020). Personal supports can be achieved through coaching from school mental health providers (e.g., social workers) and peer support, while system supports require leadership buy-in and the dedication of resources such as staff time. Second, there is a critical need for student perspectives to inform SSNOLA and all trauma-informed approaches. Engaging youth voice using methods like youth participatory action research – especially with marginalized youth in high-poverty and hypersegregated schools – can help determine if schools are meeting their needs and how trauma-informed approaches might be modified to achieve their desired impact.

### Positive Behavior Management Toolkit

#### Brief Description and Mental Health Target

The Positive Behavior Management Toolkit (PBMT; Lawson et al., 2024) is a modular set of teacher-facing resources to support K-5th grade teachers in using four core Tier 1 and Tier 2 positive behavior management practices for children with or at-risk for ADHD. The PBMT is informed by the Theory of Planned Behavior (TPB; Ajzen, 1991) and theories of habit formation (Nilsen et al., 2012), to target attitudes, norms, self-efficacy, habits, and ability to act on intentions. It was developed in partnership with teachers and other educators and designed to align with schoolwide PBIS.

#### Program Components and Theory of Change

The PBMT (see Lawson et al., 2024 for a more detailed description) uses a modular approach to support four teacher-delivered behavior management practices (e.g., behavior-specific praise) for students with or at-risk for ADHD. Each teacher-delivered practice has an extensive research base supporting its effectiveness. These practices were selected with educator input and are described using language that aligns with schoolwide PBIS. The PBMT also includes modules related to strengthening student-teacher relationships and supporting teacher wellness because these were identified as important implementation barriers and facilitators. The PBMT has three core components: (1) a library of resources, including written materials, videos, and tangible resources; (2) text-message reminders; and (3) four bi-weekly meetings with a consultant (15–20 min each), to assist the teacher with goal setting, identifying relevant resources, building motivation, problem solving, and reflecting on progress. Teachers engage with the PBMT for a period of 8 weeks. Each teacher is assigned a consultant, who could be any individual with an understanding of the school context, behavioral principles and motivational interviewing. This consultant meets with the teacher on four, brief (i.e., 15–20 min) occasions during this period to support their engagement with the PBMT resources (e.g., selecting resources that are relevant to addressing the key barriers and facilitators a teacher is experiencing). This model was determined in partnership with teachers and other educators to fit feasibly within school schedules (e.g., consultation meetings are intentionally brief and occur during teacher planning periods). Per the theory of change, the PBMT is hypothesized to increase the frequency and competence with which teachers use evidence-based Tier 1 and Tier 2 practices via strengthening their intentions and habits, which is in turn expected to lead to improvements in student behavioral outcomes.

#### Empirical Evidence of Effectiveness

To date, the PBMT has been evaluated in a small-scale pilot randomized controlled trial (Lawson et al., 2023) to examine acceptability, feasibility and preliminary observed teacher implementation outcomes (e.g., frequency and competence with which teachers use behavioral interventions) and student effectiveness outcomes (e.g., parent- and teacher- reported ADHD-related impairment). Quantitative and qualitative results, as well as observed data about teachers’ engagement with the PBMT, suggested that the PBMT was highly acceptable and feasible. The results were promising regarding the PMBT’s potential effectiveness in supporting teacher implementation outcomes but mixed regarding student outcomes. 

#### Opportunities and Challenges

The PBMT has been used primarily in the context of small-scale research studies in partnership with an urban school district, in which teachers were offered the opportunity to voluntarily enroll. Within this context, a few key challenges and opportunities have been observed. First, although all teachers using the PBMT in the pilot study attended 100% of expected consultant meetings, this required substantial flexibility from the consultant (e.g., accommodating rescheduled meetings). Similarly, to date, consultation has been provided only by a member of the research team; although school staff members (e.g., counselors, school psychologists) would be appropriate for providing consultation per the model, it would be challenging to place this additional demand on their time. However, it is notable that teachers who used the PBMT qualitatively reported that it did not increase their workload, with several teachers reported that using it lowered their workload by preventing behavioral concerns that would require time to manage. Teachers also specifically cited the PBMT’s alignment with schoolwide PBIS as making it a good fit for their needs (e.g., it supports them in doing what they are already expected to do) and as important for school leaders’ buy-in. This highlights the opportunities of teacher-delivered mental health interventions that meet teachers’ needs (e.g., preventing challenging behaviors, using expected practices) rather than impose demands unrelated to existing needs and priorities.

### Teacher Anxiety Program for Elementary Students (TAPES)

#### Program Components and Theory of Change

TAPES (Ginsburg et al., 2024) is a Tier 2, five-meeting school-home teacher delivered intervention to reduce student anxiety among elementary school students. Each conjoint teacher-parent-student meeting is approximately 20–30 min and covers specific CBT anxiety reduction strategies. The intervention is expected to be delivered over an 8–10-week period. TAPES was developed using an iterative process in collaboration with teachers, families, and school mental health experts. The components of TAPES are based on empirically supported interventions for youth with anxiety disorders (e.g., psychoeducation, relaxation training, cognitive restructuring; James et al., 2020). The key intervention component, however, is reducing avoidant behavior, often referred to as exposure. To facilitate exposure in TAPES, parental and teacher accommodation, defined as allowing (unintentionally or intentionally) a student to avoid what they are afraid of (Norman et al., 2015) is targeted directly through the use of a daily Bravery Chart which targets specific avoided situations. Students are also taught relaxation strategies and how to use coping thoughts (to counter maladaptive anxious thoughts) to facilitate exposure. The underlying theory of TAPES is that reduction in student avoidance and teacher/parental accommodation of anxiety will mediate the effects of the program on anxiety and school functioning. The use of conjoint teacher-parent-student meetings is designed to facilitate teachers’ ability to formalize mechanisms for sharing information about anxiety reduction and enhance communication between parent, teacher, and student. The school-home model has numerous advantages including fostering a collaborative approach with similar language and tools to facilitate generalization of anxiety reduction skills between school and home (Conroy et al., 2022).

TAPES includes a six-hour interactive teacher training that can be done in person or virtually. The training strategies include active/experiential learning strategies, opportunities for observation (via video clips), live modeling and role plays, and coached practice. Once teachers begin working with a student, weekly coaching is provided. Coaching is offered virtually at times convenient for teachers in an individual format. Teachers also receive an intervention manual and handouts to be used with parents and students, as well as video clips demonstrating each TAPES meeting. All training materials are provided in electronic format (and paper if preferred). The manual describes the content of each meeting, sample scripts illustrating how teachers can convey the content to students/parents effectively. A troubleshooting guide is also included in the training materials.

#### Empirical Evidence of Effectiveness

In addition to open trials (Piselli et al., 2022) TAPES was evaluated in a pilot RCT where it was compared to an active comparison condition (i.e., Teacher Anxiety Training; TAT; a three-hour didactic teacher training on student anxiety). In this study, 54 elementary teachers were randomized (1:1) blocked by grade level and were trained to deliver TAPES (*n* = 28) or TAT (*n* = 26). Fifty-four students with impairing anxiety (25 in TAPES; 29 in TAT) were enrolled. Independent evaluators completed pre-, post-, and a 3 month follow up (F3) assessments of students (for a full description of the sample, study methods and results see Ginsburg et al., 2023, 2024). Several outcomes were assessed including the feasibility of TAPES, teachers’ fidelity delivering TAPES, the impact on teachers’ knowledge and skills, and the impact on students’ outcomes. We focus here on the impact of TAPES on teachers and students. Our findings revealed that the TAPES training resulted in positive pre-post changes (with medium to large effect sizes) on teachers’ the use of anxiety reduction strategies in the classroom (Cohen’s *d* = 0.48–0.78), teachers’ confidence in their ability to implement anxiety reduction strategies (Cohen’s *d* = 0.48), on teaching self-efficacy (Cohen’s *d =* 0.46–0.69), and on reducing inappropriate accommodation of student anxiety (Cohen’s *d* = 0.85). The TAPES training, compared with the TAT training, had a significantly greater effect on teacher knowledge of student anxiety test scores (Cohen’s *d* = 2.21). In terms of student outcomes, pre-post changes *within* the TAPES sample indicated the intervention had positive effects on anxiety severity and related impairment, educational outcomes, and hypothesized mediators (i.e., behavioral avoidance, parental accommodation of anxiety), and most of these changes were maintained at the 3-month follow-up. Taken together, preliminary data demonstrate the promise of TAPES to produce positive changes in teachers and students.

#### Opportunities and Challenges

Although the outcomes of TAPES appear promising, there are several challenges to adoption and implementation. Teachers have many competing demands and carving out time to learn and practice the anxiety reduction strategies was difficult, as almost 30% who indicated interest in participating ultimately could not find the time. Another challenge was teachers’ success in identifying and enrolling students with anxiety. Finally, because teachers have limited training in addressing student anxiety, the quality of implementation was suboptimal, and additional coaching and resources to support teachers is indicated. Despite these challenges (and others) adopting TAPES also offers unique opportunities for teachers to improve their knowledge, skills, and teaching efficacy. In addition, students’ improvement in social, emotional, behavioral and academic functioning likely result in better student-teacher relationships, an improved classroom climate, and may make teachers’ job more enjoyable.

### Partners in School

#### Brief Description and Mental Health Target

The goal of Partners in School (PariS) is to ensure that parents and teachers of students with autism and co-occurring mental health challenges (in pre-K – 5th grade) are implementing evidence-based practices (EBPs) with fidelity (implemented as prescribed) at home and school, respectively, and that those EBPs are aligned (implemented in the same way) across the settings where children spend most of their time (Azad et al., 2021). Developed in partnership with principals, mental health staff, teachers, and parents, this five-step, 11-week program is designed as a Tier 2 or 3 system of support for parents and special education teachers in self-contained classrooms. See Azad et al. (2021) for full description.

#### Program Components and Theory of Change

The *PariS* consultant takes parents and teachers through the implementation process using the *Exploration*,* Preparation*,* Implementation*,* and Sustainment* (EPIS) Framework (Moullin et al., 2019). The Exploration phase consists of a pre-consultation interview in which the consultant helps prioritize the main concerns at home and at school. The Preparation phase consists of two implementation strategies – communication skills training and problem-solving consultation. First, parents and teachers complete a web-based training, *School Talk*, that presents a series of brief videos (less than 10 s) on parent-teacher communication and collaboration (Roter et al., 2012). Second, there is a consultant-facilitated problem-solving consultation meeting to develop a student EBP plan targeting the mutual concern across home and school (McGrew et al., 2016). In the *Implementation* phase, although there are 15 EBPs available for use (i.e., reinforcement, visual supports, task analysis, etc.), each student plan is usually comprised of 3–5 EBPs (e.g., setting a timer as an antecedent for transitioning, using a visual schedule to transition, and providing a sticker for reinforcement to help establish classroom routines). In *PariS*, the *same* student plan is being conducted by parents at home and by teachers at school for four weeks. All intervention materials are provided given the limited funding in schools. A pre-populated home-school note is completed daily to collect fidelity data and track the child’s progress. “Micro” coaching involves the consultant checking-in with parents and teachers after the first day of implementation, as well as a virtual meeting mid-way through the four-week implementation phase. The *Sustainment* consists of a post-consultation interview with parents and teachers separately to review student, parent, and teacher outcomes, as well as implementation. In *PariS*, we draw from the theory of planned behavior and EPIS, which suggests that attitudes, subjective norms, and perceived behavioral control influence one’s intentions, and those intentions are the best predictor of actual behavior (i.e., fidelity).

#### Empirical Evidence of Effectiveness

*PariS* has been delivered via pre-post designs and yielded promising implementation, parent/teacher, and student outcomes. Parents and teachers reported that the *PariS* problem-solving consultation and communication skills training (evaluated as separate strategies) were feasible and acceptable. It resulted in increased parent-teacher alliance as reported by parents (mean difference = 0.217, *p* < 0.001) and teachers (mean difference = 0.416, *p* < 0.001). Participants who received problem-solving consultation reported changes in specific (reduced frequency/severity of concerns) and global (reduced disruptive behaviors) child outcomes (Azad et al., 2018, 2021, 2024).

#### Opportunities and Challenges

The development and delivery of PariS is an opportunity to establish a community-academic partnership that engages partners at multiple levels. At the administrative level, engagement meetings are conducted with school leadership (principals and assistant principals) and related service providers (psychologists, counselors, etc.) about the importance of equipping parents and teachers with EBPs to address the increasing numbers of students with autism. Parents and teachers are empowered to address the common mental health challenges (e.g., behavioral challenges, anxiety) that are disruptive to home and school life. During this process, collaborative communication and problem-solving is emphasized and little prior knowledge about autism interventions is assumed, which are particularly appealing to districts since caregiver involvement and evidence-based interventions are key components of the mission statement for districts. For students, *PariS* is a unique opportunity to double the dosage, intensity, impact of EBPs for them. Nonetheless, *PariS* also presents with challenges, particularly with the translation to school-based delivery. More specifically, identifying local school-based consultants (e.g., school psychologists) to deliver *PariS* (rather than research staff) has been challenging given the time demands (phone interviews, in-person meeting, coaching) and qualifications (EBPs in autism, relationship building, conflict management) of the consultant. It is likely that aspects of the program (e.g., daily home-school fidelity data) will need to be modified to fit the roles and responsibilities of school-based mental health staff.

## Discussion

This paper highlights the opportunities and challenges of leveraging teachers to support student mental health and showcases examples of teacher-delivered programs that span a range of intervention tiers and mental health focus areas. The programs share several similarities. For example, teacher-delivered antecedent-based interventions, positive reinforcement, and approaches to communicate effectively with caregivers are common components across most of the programs. The examples also share common implementation strategies of initial training and ongoing coaching or consultation, although they vary in the intensity and time requirements of these strategies.

These examples highlight several key considerations when conceptualizing teachers as non-traditional providers of mental health interventions. First, they illustrate the importance of ensuring that teacher-delivered mental health interventions enhance teachers’ capacity within their existing roles and responsibilities (which are primarily about education, not mental health). Each of the programs described here is designed to help teachers address challenges they experience in their everyday classroom interactions (e.g., responding to student anxiety, preventing challenging behavior) that interfere with learning, with the goal of enhancing teacher capacity within their roles rather than placing additional demands on teachers. Given that teachers spend substantial time with students and impact student well-being via their day-to-day interactions (Hamre & Pianta, 2006), each of these programs targets the nature of everyday student-teacher interactions (e.g., reducing accommodation; supporting predictable routines; increasing positive reinforcement). This differs meaningfully from how traditional mental health clinicians are typically deployed to support youth mental health (i.e., through interactions that occur primarily during discrete treatment sessions). Notably, the interventions described here are not meant to replace traditional mental health interventions provided by school mental health staff (e.g., 1:1 counseling), but rather are designed to supplement and integrate with these interventions. Moreover, school mental health staff (e.g., counselors, school psychologists, social workers) could provide the coaching or implementation support to teachers in these program models. However, they will also need training, coaching, and consultation to sustain these programs within the scope of their own roles and responsibilities.

Second, the example programs described here highlight the need to provide adequate training, consultation, and other implementation support to teachers when asking them to support students’ mental health. This implementation support must account for teachers’ roles, prior training, and the school context (e.g., limited funds). Each of these programs includes an emphasis on psychoeducation for teachers and does not assume a high level of background knowledge related to mental health. They also include implementation support both at the time of initial training and ongoing technical assistance (e.g., consultation, coaching) that recognizes that teachers are not traditional mental health providers. Although the training and consultation models differ in their forms (i.e., length, format), they are each designed to be feasible within the school context (e.g., training designed to fit within staff professional development schedules, consultation designed to be flexible and fit within planning periods of the school day, providing intervention materials).

Additionally, our experiences with the example programs highlight the importance of implementation climate and leadership (e.g., principal buy-in) at the school level. SSNOLA includes an explicit focus on building organizational infrastructure (e.g., school policies and practices). Although the other case example programs operate primarily at the level of the individual classroom, school-level contextual factors and implementation support are nevertheless critical to their successful implementation. For example, during the “engagement” meetings for *PariS*, school administrators are offered different ways to be involved ranging from praising teachers for their participation, attending the parent-teacher consultation meeting, or providing teachers additional coverage when parents are only available to meet at specific days/times. These strategies were strategically chosen because they demonstrate the administrators’ commitment. This is consistent with literature about the importance of school culture and climate for the implementation of EBPs (Williams et al., 2019). Specifically, school administration can influence the availability of professional development, performance expectations, and allocation of resources and recognition (Williams et al., 2021), and are therefore well positioned to ensure that teachers receive adequate support to deliver mental health prevention and intervention programs.

Finally, our experiences with each of these example programs highlights the critical importance of including perspectives from relevant groups (e.g., teachers, school administrators, other school staff, parents, and students) in the development and implementation of teacher-delivered prevention and intervention programs. Consistent with principles that co-development with those who deliver and receive interventions leads to interventions that are more feasible and contextually relevant (Lawson & Owens, 2024), the teacher-delivered interventions described here were developed collaboratively with key partners. Accounting for key partner perspectives is particularly important to ensure that these interventions avoid potential pitfalls such as being perceived as outside of teachers’ job scope or boundaries of professional competence, exacerbating teacher burnout, or creating role confusion with school mental health professionals.

### Future Directions

Continued research is needed to further elucidate when and how teachers can effectively and appropriately be deployed to address youth mental health, and the infrastructure needed in schools to support this endeavor. In many ways, this literature is in its infancy, with many teacher-delivered interventions, including those described here, in early stages of development. evaluation, and delivery. Although not the purpose of the current paper, it is important for future research to examine teacher and student outcomes associated with teacher-delivered mental health interventions. Evaluating the effectiveness of teacher-delivered interventions is critical to advancing our understanding of whether and under what conditions teacher-delivered interventions have their intended impact.

Throughout larger-scale dissemination and evaluation of teacher-delivered interventions, it will be critical to consider the role of the school, neighborhood and geographic context, and to explicitly examine equity in implementation and effectiveness outcomes. Although there is evidence that mental health services delivered in schools can enhance access to care, particularly for students of color or low socioeconomic background (Wilk et al., 2022), there is also evidence of continued disparities in access, quality and outcomes of school mental health services (Gaias et al., 2022). Similarly, while teacher-delivered interventions may improve access for marginalized groups, they also have the potential to widen inequities if teachers in schools with fewer resources (e.g., larger class sizes, less preparatory time, limited materials) cannot support implementation. Recent implementation frameworks and recommendations (e.g., Baumann & Cabassa, 2020; Gaias et al., 2022) provide guidance for research teams on ways to explicitly consider equity in the selection of study populations, adaptation of implementation strategies, and evaluation of outcomes; these recommendations are critical for mitigating the risk that teacher-delivered interventions could widen existing inequities. Moreover, the teacher-delivered interventions described here have been implemented primarily in the elementary school context. Given high levels of unmet mental health need among adolescents (Ivey-Stephenson et al., 2020), it will be important for future research to examine whether these or other programs can be adapted for middle- or high-school contexts. However, it will be important for this work to be undertaken with a thoughtful consideration of school context, given the unique implementation considerations in high schools as compared to elementary schools (e.g., Estrapala et al., 2021).

It will also be critical for future research to identify implementation approaches that support the effective scale-up and sustainment of teacher-delivered interventions. Although we described the inherent scalability of teacher-delivered mental health interventions as an important opportunity for conceptualizing teachers as individuals who can support youth mental health, we note that the example interventions described here have predominantly been implemented in small-scale pilot studies to date with research staff providing implementation support. If these interventions have their intended impact, it will be important to scale-up beyond universities for local delivery and sustainment. For example, establishing training, supervision, and coaching packages for local school mental health staff to provide implementation support to teachers is one pathway for broader adoption. It will also be important for larger scale evaluations to assess cost and cost-effectiveness to inform decisions about scale-up. Although there is some evidence that teacher classroom management programs may be cost effective, this evidence to date is uncertain (Ford et al., 2019) and little is known about the cost effectiveness of teacher-delivered interventions with a more explicit mental health focus.

## Conclusion

Teacher-delivered mental health prevention and intervention programs hold great promise for building capacity in teachers to reach youth who may not otherwise receive mental health services, as well as meaningfully integrating mental health supports within their everyday lives. At the same time, there are important challenges to employing teachers as non-traditional mental health providers. Further research is needed to continue to unpack when and how teachers can be utilized as non-traditional mental health providers, enhance empirical evaluation of teacher-delivered interventions, and identify approaches to scale-up and sustain effective teacher-delivered prevention and intervention programs, with the ultimate goal of supporting both teachers and students.
